# Effect of 16 Weeks of Resistance Training on Fatigue Resistance in Men and Women

**DOI:** 10.2478/hukin-2014-0071

**Published:** 2014-10-10

**Authors:** Alex S. Ribeiro, Ademar Avelar, Brad J. Schoenfeld, Michele C.C. Trindade, Raphael M. Ritti-Dias, Leandro R. Altimari, Edilson S. Cyrino

**Affiliations:** 1 Londrina State University, Londrina, PR, Brazil.; 2 Maringá State University, Maringá, PR Brazil.; 3 Exercise Science Department, CUNY Lehman College, Bronx, New York, EUA.; 4 Ingá – Uningá College, Maringá, PR, Brazil.; 5 School of Physical Education, Pernambuco University, Recife, PE, Brazil.

**Keywords:** resistance training, one-repetition maximum, muscular endurance, sex

## Abstract

The purpose of this study was to investigate the effect of hypertrophy-type resistance training (RT) on upper limb fatigue resistance in young adult men and women. Fifty-eight men (22.7±3.7 years, 70.6±9.3 kg, and 176.8±6.4 cm) and 65 women (21.6±3.7 years, 58.8±11.9 kg, and 162.6±6.2 cm) underwent RT for 16 weeks. Training consisted of 10–12 whole body exercises with 3 sets of 8–12 repetitions maximum performed 3 times per week. Before and after the RT intervention participants were submitted to 1RM testing, as well as a fatigue protocol consisting of 4 sets at 80% 1RM on bench press (BP) and arm curl (AC). The sum of the number of repetitions accomplished in the 4 sets in each exercise was used to indicate fatigue resistance. There was a significant (p<0.05) time-by-group interaction in 1RM BP (men=+16%, women=+26%), however in 1RM AC no significant time-by-group interaction was observed (men=+14%, women=+23%). For the total number of repetitions, men and women showed a significant increase in BP (men=+16.3%, women=+10.5%) with no time-by-group interaction. The results suggest that the adaptation in maximal strength is influenced by sex in BP. On the other hand, for fatigue resistance, the individual’s sex does not seem to influence outcomes either in BP or AC.

## Introduction

Resistance training (RT) is a modality of physical exercise that is widely used for both performance enhancement as well as health promotion. Moreover, RT has applicability for a variety of different populations due to numerous morphological, neuromuscular, physiological, and metabolic adaptations it produces (ACSM, 2009). Among these adaptations there is an improvement in the ability to resist muscular fatigue. Fatigue resistance plays an important role in many athletic endeavors (Billaut and Bishop, 2009) and activities of daily living (Bautmans et al., 2008). In addition, a growing body of evidence suggests that a greater fatigue resistance can augment muscle hypertrophy (Schoenfeld, 2013). Also, many RT programs include a fatiguing component as a means to enhance results (Bentes et al., 2012).

The magnitude of adaptations induced by RT may be affected by a number of factors including sex, age and level of previous RT (Deschenes and Kraemer, 2002; Lemmer et al., 2007; Martel et al., 2006; Salvador et al., 2009). Sex, in particular, has been shown to significantly affect fatigue resistance variables, with most cross-sectional studies indicating that women have a greater capacity to resist fatigue than men (Hicks et al., 2001; Hunter, 2009). Direct comparisons of the effects of sex on fatigue resistance in response to RT were previously documented by Salvador et al. (2009) where women displayed a higher increase in fatigue resistance after 8 weeks of RT compared to men. However, considering that the time-course of RT affects adaptive outcomes (Gabriel et al., 2006), and given that men and women differ in various responses to RT (Hubal et al., 2005; Ivey et al., 2000; Kell, 2011; Peterson et al., 2011; Roth et al., 2001), it is possible that a longer training period may be needed to clarify sex differences in fatigue resistance. Thus, there is reason to question if differences exist between sexes with respect to fatigue resistance adaptations in response to a longer RT period. Therefore, the purpose of this study was to investigate the effect of hypertrophy-type RT on upper limb fatigue resistance comparing young adult men and women in an RT program lasting 16 weeks.

## Material and Methods

### Participants

Fifty-eight men (22.7 ± 3.7 years, 70.6 ± 9.3 kg, 176.8 ± 6.4 cm, and 22.6 ± 2.5 kg/m^2^) and 65 women (21.6 ± 3.7 years, 58.8 ± 11.9 kg, 162.6 ± 6.2 cm, and 21.6 ± 3.2 kg/m^2^) were recruited from a university population and local advertisement and then volunteered to participate in this study. All subjects completed a detailed health history questionnaire. The subjects were included in the study if they had no signs or symptoms of disease, no orthopedic injuries, were inactive or moderately active individuals (defined as performing physical activity less than twice a week), had not been regularly engaged in any RT program during the last six months before the beginning of the study, and were free from steroid use or other ergogenic aids. All participants had an adherence to training sessions >85% of the total sessions. Written informed consent was obtained from the subjects after receiving a detailed description of all procedures. This study was performed in accordance with the declaration of Helsinki, and the experimental protocol was approved by the Londrina State University Ethics Committee (Process 028/2012).

### Experimental design

The study was carried out over a period of 22 weeks. Measurements of muscular strength and fatigue resistance were performed at weeks 1–2 and 21–22. A supervised progressive RT program was performed in 2 phases each lasing 8 weeks. The first phase was carried out in weeks 3–10 and the second phase was carried out in weeks 13–20. Between phases (weeks 11–12) subjects were provided with an unloading interval designed to promote recovery and restructuring of the RT program; during these 2 weeks subjects did not perform any exercise. All sessions were performed at the same time of day, and were supervised by trained personnel. The subjects were instructed to maintain their normal level of physical activity and were specifically asked not to start a new exercise regimen during the study period.

### Anthropometry

Body mass was measured to the nearest 0.1 kg using a calibrated electronic scale (Filizola, model ID 110, São Paulo, Brazil), with the subjects wearing light workout clothing and no shoes. Body height was measured using a wooden stadiometer to the nearest 0.1 cm while subjects were standing without shoes. Body mass index was calculated as the body mass in kilograms divided by the square of the height in meters.

### Maximal muscular strength

Maximal dynamic strength was evaluated using the 1RM in the bench press (BP) and arm curl (AC), in that order at baseline and after the intervention period. The 1RM was performed with free weights in both exercises. In BP the grip was such that the thumbs were at shoulder width when the bar was resting on the support props. Complete range of motion consisted of lowering the bar until it touched the chest, and pressing it upward until locking the elbows at the top of the press. For execution of AC the subjects stood with their back against a wall to prevent any assistive motion, and the knees were positioned with a slight flexion. From a full arm-extended position, hands in supination were placed slightly wider than shoulder width and directly under the bar, which was curled using the anterior arm flexor muscles through approximately a 120° range of motion, or until the full flexion of the elbow. The rest periods between exercises ranged from 3 to 5 minutes. The test in each exercise was preceded by a warm-up set (6–10 repetitions) with 50% of the estimated load used in the first attempt of the 1RM test. The testing procedure was initiated two minutes after the warm-up. The subjects were encouraged to try to accomplish two repetitions with the imposed load in three attempts in both exercises. If the subject was successful in the first attempt, weight was added (3–10% of the first attempt load), a 3–5 min rest was given, and a second attempt was made. If this attempt was successful, a third attempt was given with an increased load (3–10% of the second attempt load), following a 3–5 min rest. If the subject was not successful in the first or second attempt, weight was removed (3–10% of the previous attempt load) and one other attempt was given. The 1RM was recorded as the last resistance lifted in which the subject was able to complete one single maximum repetition (Ritti-Dias et al., 2011). Execution technique and form of each exercise were standardized and continuously monitored to guarantee reliability of maximum strength assessment. All testing sessions were supervised by three experienced researchers for greater safety and integrity of the subjects during tests. Verbal encouragement was given on every attempt. Four 1RM sessions were performed separated by 48 hours (ICC ≥ 0.98). The highest load among the four sessions was used for analysis in each exercise. During all sessions, subjects were allowed to drink water whenever necessary and were encouraged to remain hydrated throughout testing.

### Fatigue resistance

A fatigue resistance protocol was carried out 48 hours after the 1RM session. The exercises as well as their order of performance were the same as in 1RM testing. The subjects arrived at the laboratory 2 hours after having a light meal and were instructed to avoid any caffeine and alcohol-containing beverages 48 hours before the tests. The protocol consisted of 4 sets at 80% of 1RM until voluntary exhaustion, with 2 minute rest intervals between sets and 5 minutes rest between exercises. The subjects were asked to perform a maximum number of repetitions in each set. The 2 exercises were preceded by a specific warm-up set in the same exercise used in the test, which consisted of 6–10 repetitions with approximately 50% of 1RM load of each exercise.

The fatigue index (FI) proposed by Sforzo and Touey (1996) was used to determine the drop in force output over time as calculated by the following formula:

$$\mathrm{FI}=\left[\left(S_{\left(\mathrm{first}\;\mathrm{set}\right)}-S_{\left(\mathrm{fourth}\;\mathrm{set}\right)}\right)/\left(S_{\left(\mathrm{first}\;\mathrm{set}\right)}\right)\right]*100\%$$

where: FI = fatigue index; S = Strength (load lifted x number of repetitions executed during particular sets).

### Resistance training

A supervised progressive RT program designed to induce muscular hypertrophy was performed in two 8-week phases with training carried out 3 times per week on nonconsecutive days (Monday, Wednesday, and Friday) (ACSM, 2009). All subjects were individually supervised by experienced instructors during each training session in order to reduce deviations from the study protocol and to ensure subject safety. Subjects performed RT using a combination of free weights and machines, and the exercises included total and segmental movements of upper limbs, trunk and lower limbs. The progressive RT program in the first phase consisted of 9 exercises selected to stress the major muscle groups in the following order: bench press, 45^°^-angle leg press, wide-grip behind-the-neck pulldown, leg extension, side lateral raise, supine leg curl, triceps pushdown, calf press on the leg press machine, and arm curl.

In the second phase, the RT program was altered, and 11 exercises were performed in the following order: bench press, incline dumbbell fly, wide-grip behind-the-neck pulldown, seated cable rows, seated barbell military press, arm curl, supine triceps press, leg extension, 45^°^-angle leg press, lying leg curl, and seated calf raise. After the resistance exercises, the abdominal crunch exercise was performed on the floor using the subject’s body mass, the subjects performed 3 sets, and were encouraged to perform between 50–100 repetitions in both phases.

In both phases, all subjects performed 3 sets of 8–12 repetitions maximum for all the exercises except for calf exercises (3 sets of 15–20 repetitions maximum) and were instructed to perform each repetition with a concentric-to-eccentric phase ratio of 1:2. The rest period between sets lasted 60–90 s with 2–3 min interval between each exercise. Subjects were encouraged to exert maximal effort during all sets. The training load was consistent with the prescribed number of repetitions for the three sets of each exercise. The load was adjusted weekly using the weight test for repetitions maximum as proposed by Rodrigues and Rocha (2003), which consisted of executing the first and second sets in the lower repetition zone (8 repetitions), and as many repetitions as possible in the third set. The load was adjusted according to the following equations:

$$\begin{array}{c} \mathrm{Upper}\;\mathrm{limb}\;\mathrm{exercises}:\mathrm{FW}=\mathrm{WT}+\mathrm{RE}/2\\ \mathrm{Lower}\;\mathrm{limb}\;\mathrm{exercises}:\mathrm{FW}=\mathrm{WT}+\mathrm{RE} \end{array}$$

where FW = final weight (kg); WT = weight used in the test (kg); RE = repetitions maximum performed that exceeded the lower limit (8 repetitions).

### Statistical analysis

Normality of data was checked by the Shapiro-Wilk's test. The data were expressed as mean ± standard deviation. The Levene’s test was used to analyze the homogeneity of variances. Two-way analysis of variance (ANOVA) for repeated measures was used for intra- and intergroup comparisons. In variables where sphericity was violated as indicated by the Mauchly's test, the analyses were adjusted using a Greenhouse-Geisser correction. When the *F*-ratio was significant, the Bonferroni’s *post hoc* test was applied to identify the differences. The previous RT experience between men and women was explored with an independent t-test. For all statistical analyses, significance was accepted at *p* < 0.05. The data were stored and analyzed using STATISTICA software version 7.0.

## Results

Previous RT experience was similar between men and women (14.6 ± 15.5 and 10.6 ± 16.2 months, respectively, *p* = 0.177). Changes in muscular strength are presented in Table 1. There was a significant time-by-group interaction (*p* < 0.05) in BP, in which the women had a higher relative increase than men.

Table 2 shows the maximum number of repetitions performed in 4 sets at 80% of 1RM in the BP and AC. A significant main effect of time (*p* < 0.05) was observed in the BP with no significant group-by-time interaction. The main effect of group (*p* < 0.05) was observed in the AC, in which women showed better endurance than men.

The fatigue index in the two exercises at baseline and after the RT program is presented in Table 3. A significant main effect of time (*p* < 0.05) was observed in the BP and AC, in which men and women had a similar decrease throughout the experiment.

**Figure 1:**
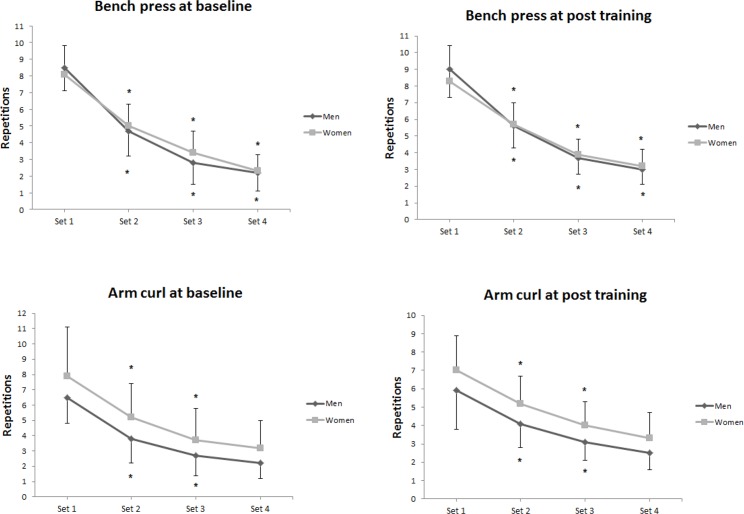
Number of repetitions performed with 80% of 1RM in bench press, and arm curl by men (n = 58) and women (n = 65) at baseline and after 16 weeks of resistance training. ^*^p < 0.05 vs. previous set. Data are expressed as mean ± SD.

Figure 1 presents a set by set analysis of the number of repetitions performed in the BP and AC at baseline and post training in men and women. A significant decrease across sets (*p* < 0.001) was observed in both sexes at baseline and after 16 weeks of the RT program.

## Discussion

The major findings of this investigation were that after 16 weeks of RT women had a higher relative increase in maximal strength than men in the BP, and sex did not influence the analyzed fatigue-related parameters.

The fatigue resistance capacity was analyzed by the maximum number of repetitions performed in a specific protocol. In this regard, we observed that men and women increased their performance after 16 weeks of RT in the BP. The mechanisms underlying the higher fatigue resistance adaptive response may be related to the specificity of energy substrate utilization. Hypertrophy-oriented RT is a model of exercise that relies heavily on glycolysis and thus produces adaptations that include an increase in glycogen storage (MacDougall et al., 1977), which may in turn mediate an improvement in multiple set exercise performance. Interestingly however, for the AC exercise no significant pre- to post-exercise changes were seen in the number of repetitions performed at 80% of 1RM. A possible explanation for this difference between exercises may be related to the order in which training was carried out. While the BP was always positioned at the beginning of the training session, the AC was the last upper limb exercise performed. Thus, arm flexor muscles always acted under pre-fatigued conditions, since the agonist muscles for AC execution were activated as antagonists and synergists in previous exercises. However, it is important to note that the exercise order applied in this study was performed according to literature recommendations (ACSM, 2009). Moreover, it could be argued that an increased level of fatigue experienced in the elbow flexors during training should have resulted in greater adaptive response with respect to the hypertrophy process. This finding warrants further investigation.

The increase in number of repetitions performed observed in our experiment does not agree with a previous study conducted by Salvador et al. (2009), who found greater enhancements in women compared to men. These conflicting results may be related mainly to the time of RT used in the two studies, since the former investigated a shorter period of intervention (8 weeks) compared to ours. Taken together, these results show that the time-course promotes an important impact on RT outcomes. In this regard, our results actually indicate a different outcome when the training period is extended. Another important feature of our investigation was a large number of participants. This provided a high degree of statistical power, thereby increasing confidence in the ability to draw valid conclusions from results.

Another important factor that may play an important role in fatigue resistance adaptations, may be the individual’s training status. For example, Willems et al. (2012) investigated 12 weeks of fatiguing RT in men with at least one year of previous training experience. Subjects were randomized to receive either a supplement containing creatine monohydrate, whey protein, glutamine and HMB or a placebo. Results showed that the placebo group increased bench press to fatigue at 80% 1RM by 50% after 12 weeks of training. Conversely, our study found an increase in 80% 1RM bench press muscular endurance of 16.3% and 10.5% in men and women, respectively, after 16 weeks of training. These results would seem to suggest that experience in RT improves the response to a fatiguing bench press protocol. It should be noted that our study investigated muscular endurance over 4 sets separated by 2 minutes rest while that of Willems et al. (2012) employed a single set trial. Further research is required on this topic to elucidate the differences between trained and untrained subjects.

Most cross-sectional studies indicate that women have greater capacity to resist fatigue compared to men (Hunter, 2009). This is believed to be related to several factors including differences in the amount of skeletal muscle mass, energy substrate utilization, muscle morphology, and/or neuromuscular activation (Avin et al., 2010; Hicks et al., 2001; Hunter, 2009). Our results highlight that these acute differences in dynamic exercises may be task-dependent since women were found to exhibit different fatigue characteristics than men only in the BP.

In all fatigue resistance protocol exercises we observed decreases in the FI over the experimental period with no significant interaction. Our results confirmed the responses reported in a previous study that evaluated the FI (Salvador et al., 2009), although the possible explanations for these modifications (especially anaerobic metabolism adaptations) require further investigation. Nevertheless, improvement of the FI with adjusted-loads over time in our study should be considered a positive adaptation in response to progressive RT because it was associated with increased training overload. Considering that most resistance training practitioners perform multi-set protocols, we sought to analyze the capacity to sustain repetitions for an exercise over repeated sets. Consistent with the previous results observed in young men and women (Eches et al., 2013; Salvador et al., 2009), we found that fatigue impeded the ability to sustain workload over multiple sets of the same exercise with 2 minute rest periods.

The results of this study indicate that regional changes in maximal muscular strength in response to 16 weeks of RT may be influenced by sex, in which the relative increases in BP 1RM were higher in women but absolute changes were larger in men. These findings agree with previous studies that also found greater relative 1RM gains in women (Hubal et al., 2005; Lemmer et al., 2007; O'Hagan et al., 1995; Peterson et al., 2011), as well as that the influence of sex on changes in muscular strength is task dependent (Lemmer et al., 2001; Lemmer et al., 2007). The mechanisms underlying the differences between sexes are not well understood. It has been hypothesized that several factors may account for these differences. The greater relative increase in maximal muscular strength in women compared to men might be related to a possible difference in neuromuscular control response between the sexes (Hatzikotoulas et al., 2004; Lemmer et al., 2000). In this regard, it is noteworthy that relative strength increases are higher in women but relative hypertrophy may be higher in men (Hubal et al., 2005; Ivey et al., 2000; Peterson et al., 2011), suggesting that neural adaptations to weight training are greater in women. Furthermore, not all mechanisms are necessarily physiological. For example, the differences also could have resulted from exercise familiarity, because although there was no sex difference in previous RT experience, women are often more motivated to train the lower body musculature at the expense of the muscles of the trunk and arms compared to men. Thus, it is possible that the male subjects could have been more familiar with the BP and the women, therefore, may have been in a position to derive greater strength gains over the course of the study given that neuromuscular adaptations from RT programs are greater in less-trained individuals compared with well-trained (Deschenes and Kraemer, 2002). However, other studies have provided conflicting evidence about possible sex-related responses in muscular strength after a RT program (Abe et al., 2000; Martel et al., 2006; Ritti-Dias et al., 2005; Salvador et al., 2009). Nevertheless, because there are differences with respect to methodological procedures among studies such as varying intensities, durations, and volumes, it is difficult to draw consistent conclusions regarding responses of specific muscular strength adaptations, since these factors may affect the degree and rate of response.

An important factor that may influence the maximal strength results is the 1RM test familiarization. Previous studies have shown that adequate evaluation of maximal strength requires conducting familiarization sessions for the 1RM until stabilization of the load lifted is achieved (Ritti-Dias et al., 2011; Soares-Caldeira et al., 2009). Without such familiarization sessions there is a strong possibility of underestimating maximal strength. Given this finding, we believe that the four 1RM trial applied in our experiment is a strong point of our study. Other study strengths include rigid standardization of the training protocols in both stages of the study, the use of progressive overload, weekly monitoring of training load, and a large number of participants. Nevertheless, the study also has some limitations. The protocol used to assess fatigue resistance only allowed determination of the decrease in performance analysis. The determination of the possible mechanisms involved needs more objective methods such as electromyography, biochemical indicators, and muscle biopsy. The data found in our study are limited to the muscle groups and the specific exercises analyzed, thus we cannot rule out the possibility that different results may manifest from the performance of lower body exercises. However, the findings of our experiment advances the knowledge of the RT adaptations related to sexual dimorphism. Future research should seek to determine possible mechanistic explanations for these findings.

In conclusion, our results suggest that the adaptation in maximal strength is influenced by sex, in which women have a better relative response in BP exercise. On the other hand, for fatigue resistance, sex does not influence adaptations either in the BP or AC when resistance training is carried out over a period of 16 weeks. These findings refute previous research showing that women have greater fatigue-resistance compared to men.

## Figures and Tables

**Table 1:** One-repetition maximum test (kg) at baseline and post-training (16 weeks) in men and women

	Men (n = 58)	Women (n = 65)	ANOVA	*F*	*p*
Bench press					
Pre	68.7 ± 16.0§	29.2 ± 6.3	Group	378.14	< 0.001
Post	79.8 ± 15.4^§*^	36.9 ± 6.9^*^	Time	820.58	< 0.001
Δ%	+16.2	+26.4	Interaction	27.42	< 0.001
Arm curl					
Pre	40.0 ± 7.0^§^	21.7 ± 3.8	Group	358.12	< 0.001
Post	45.5 ± 6.8^§*^	26.6 ± 4.1^*^	Time	547.01	< 0.001
Δ%	+13.8	+22.6	Interaction	2.29	0.132

^*^ p < 0.05 vs Pre.

^§^ p < 0.05 vs. women. Data are expressed as mean ± SD.

**Table 2:** Total number of repetitions in four sets with 80% of 1RM at baseline and post-training (16 weeks) in men and women

	Men (n = 58)	Women (n = 65)	ANOVA	*F*	*p*
Bench press					
Pre	18.4 ± 4.5	19.0 ± 4.3	Group	0.03	0.825
Post	21.4 ± 4.2^*^	21.0 ± 4.3^*^	Time	24.84	< 0.001
Δ%	+16.3	+10.5	Interaction	0.90	0.343
Arm curl					
Pre	15.4 ± 4.6^§^	20. 2 ± 8.1	Group	34.39	< 0.001
Post	15.8 ± 4.7^§^	19.7 ± 4.9	Time	0.02	0.887
Δ%	+2.6	−2.5	Interaction	0.56	0.453

^*^ p < 0.05 vs Pre.

^§^ p < 0.05 vs. women. Data are expressed as mean ± SD.

**Table 3:** Fatigue index (%) at baseline and post-training (16 weeks) in men and women

	Men (n = 58)	Women (n = 65)	ANOVA	*F*	*p*
Bench press					
Pre	74.2 ± 11.8^§^	70.5 ± 12.1	Group	6.74	0.01
Post	65.7 ± 11.4^§*^	59.8 ± 15.9^*^	Time	42.53	< 0.001
Δ%	−11.5	−15.2	Interaction	0.50	0.480
Arm curl					
Pre	64.2 ± 16.3^§^	54.4 ± 21.1	Group	5.26	0.02
Post	54.8 ± 20.7^§*^	49.7 ± 24.3^*^	Time	5.54	0.02
Δ%	−14.6	−8.6	Interaction	0.65	0.421

^*^ p < 0.05 vs Pre.

^§^ p < 0.05 vs. women. Data are expressed as mean ± SD.
